# Atomic Layer Deposition of Nickel Oxides as Electrocatalyst for Oxygen Evolution Reaction

**DOI:** 10.3390/nano15070474

**Published:** 2025-03-21

**Authors:** Jueyu Chen, Ruijie Dai, Hongwei Ma, Zhijie Lin, Yuanchao Li, Bin Xi

**Affiliations:** School of Materials Science and Engineering, Key Laboratory for Polymeric Composite and Functional Materials of Ministry of Education, Sun Yat-sen University, Guangzhou 510006, China; chenjy556@mail2.sysu.edu.cn (J.C.); dairj6@mail2.sysu.edu.cn (R.D.); mahw@mail2.sysu.edu.cn (H.M.); linzhj39@mail2.sysu.edu.cn (Z.L.)

**Keywords:** atomic layer deposition, nickel oxides, oxygen evolution reaction, nanocatalyst

## Abstract

In this study, we present atomic layer deposition (ALD) of nickel oxides (NiO_x_) using a new nickel precursor, (methylcyclopentadienyl)(cyclopentadienyl)nickel (NiCp(MeCp)), and ozone (O_3_) as the oxygen source. The process features a relatively short saturation pulse of the precursor (NiCp(MeCp)) and a broad temperature window (150–250 °C) with a consistent growth rate of 0.39 Å per cycle. The NiO_x_ film deposited at 250 °C primarily exhibits a polycrystalline cubic phase with minimal carbon contamination. Notably, the post-annealed ALD NiO_x_ film demonstrates attractive electrocatalytic performance on the oxygen evolution reaction (OER) by providing a low overpotential of 320 mV at 10 mA cm^−2^, a low Tafel slope of 70.5 mV dec^−1^, and sufficient catalytic stability. These results highlight the potential of the ALD process using the NiCp(MeCp) precursor for the fabrication of high-activity catalysts.

## 1. Introduction

The oxygen evolution reaction (OER) plays a critical role in water splitting for sustainable hydrogen production to address the growing energy crisis [1], but its efficiency is limited by intrinsic sluggish kinetics due to the large dynamic barrier associated with the four-electron transfer mechanism [2,3]. Noble metal-based electrocatalysts, particularly iridium and ruthenium oxides, exhibit exceptional performance in mitigating the kinetic barrier with a relatively low overpotential [4,5]. However, their scarcity and high cost significantly hinder the large-scale commercial applications, thereby driving extensive research efforts toward developing cost-effective OER catalysts based on earth-abundant elements, such as transition metal oxides [6]. Among these alternatives, nickel oxide (NiO_x_)-based electrocatalysts have emerged as promising candidates for enhancing the OER process, combining economic viability with impressive catalytic activity, particularly under alkaline conditions. Pioneering studies have demonstrated that NiO_x_-based catalysts could exhibit nearly ideal anodic electron-transfer coefficients and excellent electrochemical activity [7,8]. Additionally, nickel oxides have remarkable versatility, finding a wide range of applications in other scientific and technological fields, including photoelectrochemical water splitting [9,10], p-type semiconducting devices [11,12], perovskite solar cells [13], and electrochromic devices [14].

More recently, various strategies have been developed to further enhance the OER activity of nickel oxides, including tuning their size [15], microstructure [16], and composition [17]. Among these, fabricating nickel oxides with optimized film properties through various techniques is an essential approach, including solution combustion synthesis [18], sol–gel method [19], pulsed laser deposition [20,21], physical vapor deposition [12,22], etc. However, these conventional methods often result in films with rough, porous, and inhomogeneous surfaces. In contrast, atomic layer deposition (ALD) is a widely used technique for the fabrication of high-quality thin films, owing to its precision control over film thickness, homogeneity, and conformality [23,24]. Particularly, the ability of ALD to deposit conformal coatings on substrates with complex three-dimensional geometries makes it advantageous for electrocatalytic applications [25,26,27,28].

Nickel precursors with cyclopentadienyl (Cp) ligands are generally used to prepare ALD-NiO_x_ films for the favorable volatility and cost-effectiveness, including bis(cyclopentadienyl)nickel (Ni(Cp)_2_) [13,29], bis(methylcyclopentadienyl)nickel (Ni(MeCp)_2_) [30,31], and bis(ethylcyclopentadienyl)nickel (Ni(EtCp)_2_) [32,33]. However, these precursors typically require co-reactants with high oxidation reactivity (e.g., O_2_ plasma and O_3_), and the ALD processes often exhibit relatively slow reaction kinetics, leading to significant carbon contamination in the deposited films [34]. Recent advances in precursor development have introduced alternative compounds, including bis(1,4-di-tert-butyl-1,3-diazadienyl)nickel [11] (Ni(^tBu2^DAD)_2_) and bis(N,N′-di-tert-butylacetamidinato)nickel [35] (Ni(AMD)_2_), which demonstrate excellent film growth rates and minimal impurity incorporation, but their elevated costs contradict the abovementioned requirements of the OER catalysts. These limitations highlight the need for developing novel nickel precursors with cost efficiency, high reactivity, and optimal film quality in ALD growth aimed at OER enhancement.

Herein, we demonstrate a new ALD process for NiO_x_ using (methylcyclopentadienyl)(cyclopentadienyl)nickel (NiCp(MeCp)) as the nickel precursor, which exhibits relatively fast reaction kinetics with ozone (O_3_) as the oxygen source. The focus of this work is on the development of the ALD process and the comprehensive characterization of the resulting NiO_x_ films, including surface morphology, film composition, phase structure, and electrical/optical properties. In addition, catalytic performances of the ALD NiO_x_ films are explored to demonstrate their application potential in OER.

## 2. Materials and Methods

**Figure 1:**
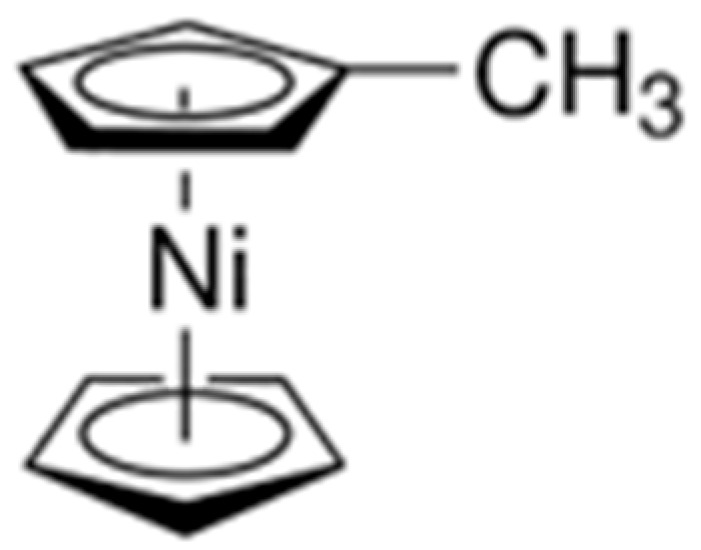
Structure of the NiCp(MeCp) precursor.

Prior to deposition, all substrates (Si, SiO_2_, sapphire, glass, and stainless steel mesh) were ultrasonically cleaned with acetone, ethanol, and deionized water in sequence, followed by nitrogen gas drying. NiO_x_ films were deposited using a showerhead commercial T-ALD reactor (Picosun R-200 Standard, Espoo, Finland) with NiCp(MeCp) (dark green powder, 99%, invented by Suzhou Origin Materials Technology Co., Ltd., Suzhou, China) as the Ni precursor and O_3_ as the oxidant. The precursor NiCp(MeCp) (Figure 1) was heated at 80 °C for continuous vapor supply during deposition (1 torr at 71 °C). O_3_ was produced by an ozone generator (GUOLIN CF-G-3-30G, Qingdao, China) under an electric current of 1.5 A. High-purity nitrogen (N_2_, 99.999%) was used as the carrier gas and purging gas, with a flow rate of 100 sccm. A single cycle of the ALD process consists of 2 s pulse of NiCp(MeCp), 5 s of N_2_ purge, 5 s pulse of O_3_, and 5 s of N_2_ purge.

The thickness of the ALD NiO_x_ films was measured using ellipsometry (J. A. Woollam Co. alpha-SE, Lincoln, OR, USA) with an incidence angle of 70°. Measurements were taken in a minimum of four different locations over 380–890 nm using a Cauchy optical model, and the average film thickness was divided by the number of deposition cycles to yield the growth rate (GPC). The top-view surface morphology was characterized by scanning electron microscopy (SEM, ZEISS Sigma 300, Oberkochen, Germany) and atomic force microscopy (AFM, Bruker Dimension Fastscan, Karlsruhe, Germany) in the PeakForce Tapping mode. Connected with an energy dispersive spectrometer (EDS), SEM was utilized to observe cross-sectional microstructure and elements. The chemical composition of NiO_x_ films was analyzed using X-ray photoelectron spectroscopy (XPS, Thermo Scientific K-Alpha, Waltham, MA, USA, Al Kα X-ray source) after removal of surface oxides and carbon via surface etching for 17 s (2 keV Ar^+^). For XPS depth profile, 1 keV Ar^+^ ions were used for sputtering at different film depths. The crystal structure information was obtained through grazing incidence X-ray powder diffraction (GIXRD, Rigaku Smartlab 9 kW, Tokyo, Japan, Cu Kα X-ray source), and the samples were irradiated at 0.5° in the 2θ range of 30–90° with a step size of 0.02°. The electrical and optical properties were evaluated with a Hall effect test system (JouleYacht HET-RT, Wuhan, China) and an ultraviolet-visible spectrophotometer (UV-Vis-NIR, Agilent Cary 5000, Santa Clara, CA, USA), respectively.

The OER catalytic performance was tested on a three-electrode electrochemical workstation (CHI660E, CH Instrument Ins., Shanghai, China) utilizing a Hg/HgO electrode as the reference and a calibrated graphite rod as the counter electrode. All samples were fabricated as working electrodes (1.0 cm × 1.0 cm). The reported potentials were measured in 1.0 M KOH and calibrated to the reversible hydrogen electrode (RHE) using E_RHE_ = E_Hg/HgO_ + 0.098 + 0.059 × pH. Linear scan voltammetry (LSV) curves of OER tests were collected from 0.9 V to 2.0 V (vs. RHE) at a scan rate of 5 mV s^−1^ in 1.0 M KOH, and the LSV data were collected at the second sweep. For compensating the voltage loss caused by the electrolyte solution, the curves were 85% Ohmic (iR) drop corrected. The overpotential (η) was derived from the difference between the potential at the specific current density in LSV curves and the equilibrium potential (1.23 V). Tafel analysis was performed to evaluate the OER reaction kinetics. Electrocatalytic stability was assessed under cyclic voltammetry (CV) cycles and continuous working time. The electrochemical surface area (ECSA) was calculated by means of electrochemical double-layer capacitance (C_dl_) through the implementation of varying scan rates during CV curves. Electrochemical impedance spectroscopy (EIS) curves were obtained under identical experimental conditions to estimate the resistance for iR compensation. All samples for OER tests were deposited on the stainless steel (SS) mesh substrate, which features low price and efficient OER catalytic performance [36].

## 3. Results and Discussion

### 3.1. ALD Process Development

**Figure 2:**
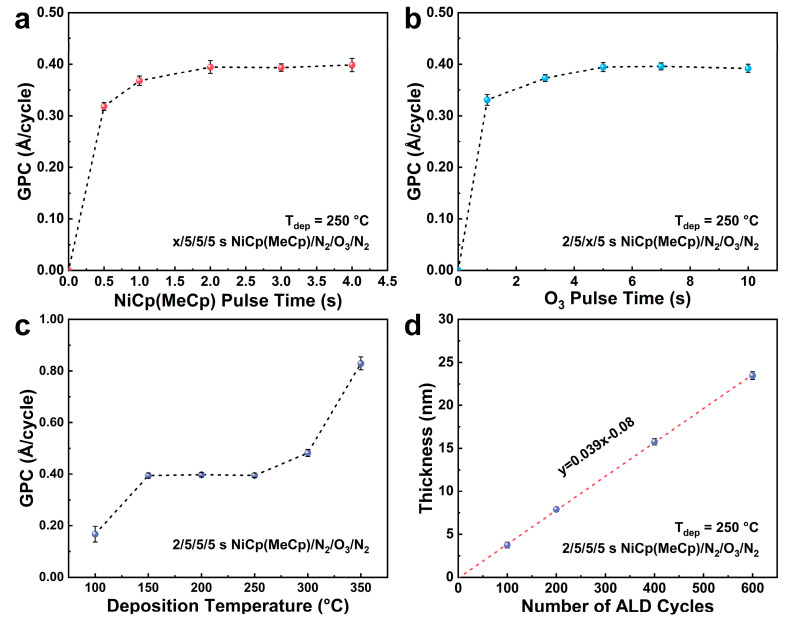
Saturation behavior of the NiO_x_ film deposition at 250 °C by varying the pulse lengths of (**a**) NiCp(MeCp) and (**b**) O_3_; (**c**) GPC of the NiO_x_ film as a function of substrate temperature; (**d**) the NiO_x_ film thickness versus the number of ALD cycles.

In a typical ALD process, self-limiting reactions take place at the substrate surface, which allows the preparation of uniform, conformal, and high-quality thin films [23]. A key indicator of self-limiting ALD behavior is the saturation of growth rate as a function of the precursor pulse length within a specific temperature range [37]. In a growth rate study for the NiCp(MeCp) precursor, the deposition was performed at 250 °C (T_dep_) for 200 cycles with the pulse length of O_3_ co-reactant set to 5 s. As shown in Figure 2a, the growth per cycle (GPC) of the NiO_x_ thin films gradually increased with increasing pulse length of NiCp(MeCp) and levelled off at 2 s with a corresponding GPC of 0.39 Å/cycle, indicating the saturation of the Ni precursor pulse. To study the saturation behavior for the co-reactant O_3_, the pulse length of NiCp(MeCp) was set to 2 s, and other parameters remained the same. Figure 2b shows that the O_3_ pulse saturated at 5 s. These results confirmed the self-limiting growth in the ALD process. It needs to be noted that NiCp(MeCp) can reach saturation on the Si surface within 2 s compared to other Ni precursors with Cp ligands that typically saturate at longer pulse times. Hufnagel et al. [13] investigated the ALD process using Ni(Cp)_2_ and O_2_ plasma and found that three precursor pulses and 90 s of plasma exposure were needed to achieve precursor saturation in an ALD cycle. The work by Barr [33] also mentioned that total pulse and exposure time of Ni(EtCp)_2_ was set as 12 s finally with a GPC of 0.028 nm/cycle. Given the asymmetric structure of NiCp(MeCp) with one Cp ring bearing a large methyl substituent, it is speculated that spatial asymmetry and steric hindrance of the substituent groups facilitate dissociation of the ligands as well as binding to the surface during chemisorption [38].

To identify the temperature range (ALD window) for self-limiting growth of NiO_x_ films, the substrate temperature was elevated from 100 °C to 350 °C, and the pulse lengths of NiCp(MeCp) and O_3_ were set to 2 s and 5 s, respectively. As shown in Figure 2c, the growth rate exhibits a wide ALD window between 150 °C and 250 °C, where the GPC remains constant at 0.39 Å/cycle. Reduced growth rate is observed below 150 °C, probably due to insufficient precursor adsorption on the surface. Above 250 °C, the growth rate increases, which could be ascribed to thermal decomposition of the precursor [23,37]. In addition, Figure 2d reveals linear growth of the NiO_x_ film as a function of the number of ALD cycles with the slope closely matching the GPC value obtained from the saturation curves, which confirms that the deposition process follows typical ALD behavior using NiCp(MeCp) and O_3_. Notably, minimal nucleation delay of two cycles is observed, suggesting that a high nucleation density is achieved in the initial cycles, allowing for the rapid formation of a continuous film [39].

### 3.2. Characterization of ALD NiO_x_ Films

**Figure 3:**
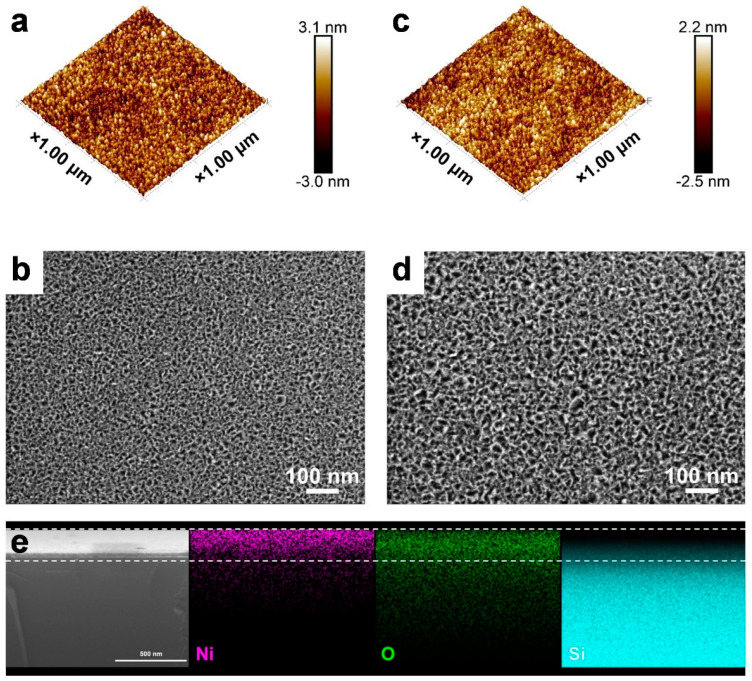
(**a**) AFM and (**b**) SEM images of the as-deposited ALD NiO_x_ films. (**c**) AFM and (**d**) SEM images of the post-annealed ALD NiO_x_ films. (**e**) Cross-sectional SEM and EDS mapping of a 30 nm NiO_x_ film.

To analyze the surface morphology and assess the surface roughness, AFM and SEM were employed for the ~20 nm thick NiO_x_ films in both as-deposited and post-annealed states. The as-deposited NiO_x_ refers to the pristine ALD NiO_x_ film deposited at 250 °C, while the post-annealed NiO_x_ was subjected to in situ O_3_ treatment at 350 °C for 30 min. As depicted in Figure 3a, the as-deposited sample exhibits a homogeneous surface with small and uniformly distributed particles, with a root mean square roughness (R_q_) of ~0.623 nm. The nanoparticle-like surface features are also confirmed by the SEM image (Figure 3b). After post-annealing, the crystallite grains became substantially larger with more distinct and well-defined nano-crystallites observed in Figure 3c,d, while the roughness (R_q_) slightly increased to 0.655 nm. It is noted that increasing deposition temperature results in bigger particles and higher roughness (Figures S1 and S2), highlighting that the deposition temperature also influences the surface morphology of NiO_x_ films. Additionally, Figure 3e shows a uniform distribution of Ni and O elements in the cross-sectional EDS mapping, further confirming the fabrication of uniform NiO_x_ thin films.

The elemental composition of NiO_x_ samples deposited using 400 ALD cycles at various deposition temperatures (i.e., 150, 200, and 250 °C) was analyzed via XPS after Ar^+^ sputter surface etching for 17 s to examine the composition across the ALD window. The results are summarized in Table 1. The Ni/O ratio was found to be in the range of 1.1 to 1.2 at all deposition temperatures, but the films are expected to have a Ni/O ratio much closer to unity due to the preferential sputtering (discussed later). It needs to be noted that the carbon (C) content of the as-deposited films decreases with increasing deposition temperature, which is primarily attributed to the enhanced decomposition of organic ligands at higher temperatures. Moreover, the ALD NiO_x_ sample that was deposited at 250 °C followed by in situ O_3_ annealing treatment at 350 °C for 30 min exhibited a slight reduction in the C impurity content.

**Figure 4:**
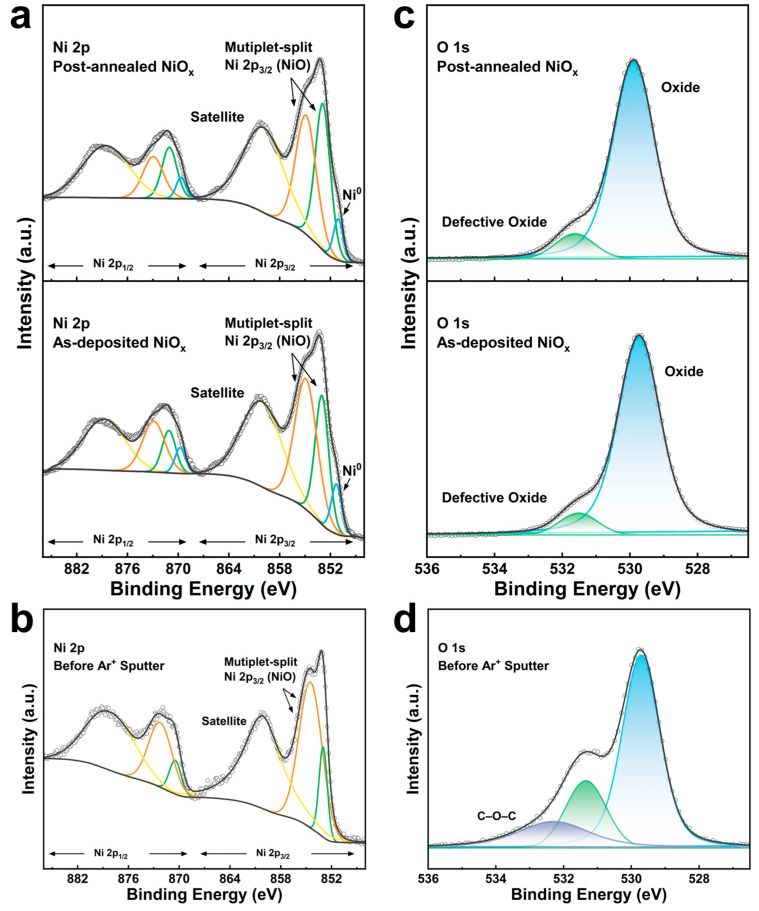
(**a**) XPS spectra of Ni 2p of the post-annealed (**top**) and the as-deposited (**bottom**) ALD NiO_x_ films after Ar^+^ sputter surface etching. (**b**) XPS spectrum of Ni 2p of the as-deposited NiO_x_ film before Ar^+^ sputter surface etching. (**c**) XPS spectra of O 1s of the post-annealed (**top**) and the as-deposited (**bottom**) ALD NiO_x_ films after Ar^+^ sputter surface etching. (**d**) XPS spectrum of O 1s of the as-deposited NiO_x_ film before Ar^+^ sputter surface etching.

High-resolution XPS spectra of Ni 2p and O 1s for both the as-deposited and post-annealed ALD NiO_x_ films at T_dep_ of 250 °C are presented in Figure 4. The Ni 2p spectrum (Figure 4a) was deconvoluted into the 2p_3/2_ and 2p_1/2_ components. The Ni 2p_3/2_ region for both the as-deposited and post-annealed NiO_x_ films displayed two prominent peaks at 853.1 eV and 855.0 eV, corresponding to the inherent multiplet splitting of the Ni^2+^ oxidation state in NiO [40,41], along with a broad satellite peak at higher binding energy. Interestingly, a Ni metal peak (Ni^0^) was detected at 851.3 eV in the Ni 2p spectrum of the as-deposited ALD NiO_x_ film [42], but no such peak was observed before sputtering (Figure 4b). This metal peak may be attributed to the preferential sputtering of oxygen during the Ar^+^ surface cleaning process [42,43,44]. As shown in Figure 4c, the O 1s spectra consist of two peaks at 529.9 and 531.6 eV for the as-deposited NiO_x_ and 529.7 and 531.5 eV for the post-annealed NiO_x_, which are assigned to the NiO lattice oxygen and defective sites within the oxide [45], respectively. In addition, a distinct C–O–C peak was detected before the Ar^+^ etching treatment (Figure 4d), while the C 1s peak disappeared after Ar^+^ sputtering (Figure S3), indicating that the NiO_x_ film surface is prone to the adsorption of airborne contaminants. This implies that the NiO_x_ film exhibits strong adsorption energy, making it an effective heterogeneous catalyst.

**Figure 5:**
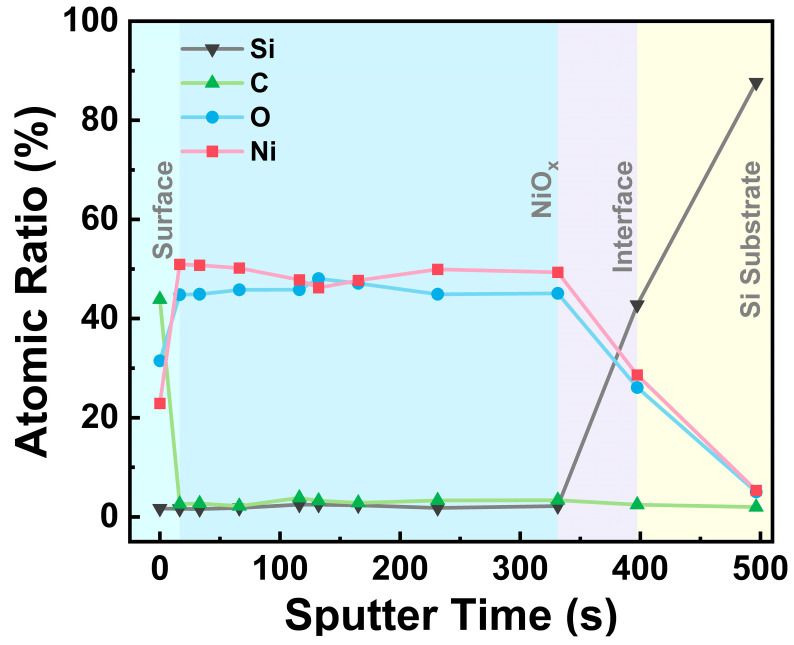
XPS elemental depth profile for the as-deposited NiO_x_ film.

The elemental distribution of the NiO_x_ film by etch depth was further explored by XPS elemental depth measurement in the as-deposited NiO_x_ film (~50 nm) on Si substrate. The depth profiling (Figure 5) showed a constant composition throughout the film with the major constituting elements of Ni and O, indicating that the NiCp(MeCp) precursor and O_3_ reacted completely throughout the deposition process. The C impurities are largely concentrated on the film’s surface with a relatively low content inside the film, suggesting surface contamination, which agrees with the XPS spectra before and after sputtering (Figure 4). At the interface between the NiO_x_ film and the Si substrate, the ratios of Ni and O atoms fall, while the ratio of Si atoms rises.

**Figure 6:**
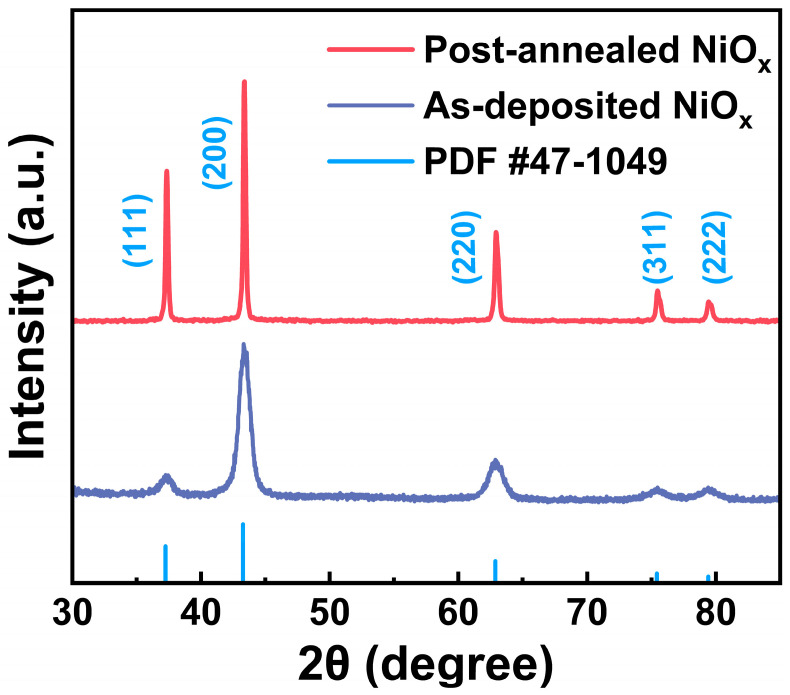
GIXRD patterns of the as-deposited and post-annealed NiO_x_ samples.

GIXRD analysis was employed to investigate the crystal structure of both the as-deposited and post-annealed NiO_x_ samples on Si(100). As shown in Figure 6, the diffraction peaks matched the reference pattern (PDF #47-1049), confirming a cubic rock-salt NiO_x_ crystal structure for both samples. No peaks attributable to the metal Ni phase were observed. The diffraction patterns revealed that (200) was the preferred crystallographic orientation for both NiO_x_ samples. Compared to the as-deposited sample, the post-annealed sample exhibited a significant increase in diffraction intensity, especially for the (111) peak, indicating an enhancement in crystallinity [46]. Crystallite sizes were calculated using the Scherrer equation [47], yielding an approximate size of ~40 nm with (200) orientation for the post-annealed NiO_x_, compared to ~10 nm for the as-deposited NiO_x_.

Room temperature Hall measurements were conducted to determine the resistivity of ~22 nm NiO_x_ films deposited on sapphire substrates. Compared to the as-deposited film with a resistivity of 7.91 × 10^2^ Ω cm, the resistivity of the annealed film decreased to 1.37 × 10^2^ Ω cm, which is in good agreement with previous reports [30,48]. Additionally, the optical properties of the as-deposited and post-annealed NiO_x_ films were investigated using UV–Vis spectroscopy (Figure S4), and the results demonstrated that both films exhibited an excellent transmittance of ~90% in the visible region with a direct optical bandgap of ~3.5 eV, which is consistent with previous reports [49,50].

### 3.3. Catalytic Performance on OER

**Figure 7:**
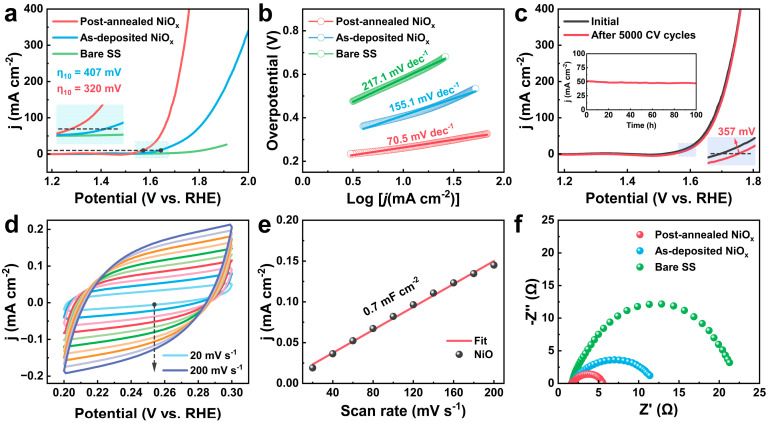
Catalytic performances on OER. (**a**) Evaluation of OER performance by LSV and (**b**) Tafel slopes for different samples. (**c**) OER polarization curves for the post-annealed NiO_x_ film before and after 5000 CV cycles. The insert reports 100 h long-term stability via chronoamperometric measurement. (**d**) CV measurement at different scan rates (20–200 mV s^−1^) and (**e**) double-layer capacitance derived from the CV curves for the post-annealed NiO_x_ film. (**f**) EIS Nyquist plots.

To evaluate the catalytic performances of the as-deposited and post-annealed NiO_x_ films, uniform 15 nm thick films were deposited on stainless steel (SS) mesh substrates and assessed for OER activity in 1.0 M KOH electrolyte using linear scan voltammetry (LSV) in a standard three-electrode system. As shown in Figure 7a, the post-annealed NiO_x_/SS exhibited significantly enhanced performance with an overpotential (η) of 320 mV, compared to the as-deposited NiO_x_/SS with η = 407 mV at a current density (j) of 10 mA cm^−2^. Furthermore, the Tafel slopes for the post-annealed NiO_x_/SS, the as-deposited NiO_x_/SS and bare SS were 70.5, 155.1, and 217.1 mV dec^−1^, respectively (Figure 7b), indicating that the annealing treatment also facilitates the OER kinetics. These enhancements in the catalytic activity of the post-annealed NiO_x_/SS can be associated with the change in crystallinity [51] and the increased fraction of crystal plane (111) that corresponds to a larger hydroxide coverage than (200) [25,52].

The electrochemical stability of the post-annealed NiO_x_/SS was systematically evaluated through comprehensive cyclic voltammetry (CV) and chronoamperometry measurements. Figure 7c shows that the η increased slightly to 357 mV at a current density of 10 mA cm^−2^ after 5000 CV cycles scanning from 1.2 to 1.8 V (relative to RHE) at a scan rate of 5 mV s^−1^. Furthermore, after a chronoamperometry test conducted at 1.64 V (vs. RHE), the post-annealed NiO_x_/SS maintained a current density of ~50 mA cm^−2^ for 100 h (Figure 7c inset), and there is no significant degradation of catalyst performance. These results demonstrate that the post-annealed NiO_x_/SS electrode possesses excellent long-term electrochemical stability. Compared with the previously reported NiO_x_-based catalysts prepared by different methods (Table S1) [9,13,53,54,55,56], our post-annealed NiO_x_/SS catalyst also exhibits better OER performance with reduced η and improved catalyst stability in alkaline solution. From the CV data recorded at scan rates ranging from 20 to 200 mV s^−1^ in the non-Faradaic region (Figure 7d), the electrochemical surface area (ECSA) was calculated using the electrochemical double-layer capacitance (C_dl_) method. The post-annealed NiO_x_/SS exhibited a high C_dl_ of 0.7 mF cm^−2^ (Figure 7e), indicating a large density of catalytic active sites [57]. Additionally, electrochemical impedance spectroscopy (EIS) revealed the shortest semicircle with the smallest charge transfer resistance for the post-annealed NiO_x_/SS sample (Figure 7f), suggesting enhanced reaction kinetics and efficient charge transfer processes at the electrode interface. Collectively, these results demonstrate that the post-annealed NiO_x_ film showed great catalytic performance on OER, with a low overpotential, a large active surface area, fast kinetics, and excellent long-term stability.

## 4. Conclusions

We have developed a new ALD process for producing high-quality NiO_x_ films using NiCp(MeCp) as the precursor and O_3_ as the co-reactant. This approach achieved relatively short saturation pulses for the asymmetric precursor with a GPC of 0.39 Å per cycle. The as-deposited films exhibited excellent properties, including high purity with minimal carbon contamination, good crystallinity in the cubic phase, and favorable electrical and optical characteristics. After O_3_ annealing, the films demonstrated outstanding catalytic performance for OER, with a low overpotential of 320 mV and a Tafel slope of 70.5 mV dec^−1^, highlighting their enhanced electrochemical activity and stability. Overall, the results indicate that the ALD process using NiCp(MeCp) precursor is a promising method for fabricating high-quality NiO_x_ films with excellent OER catalytic performance.

## Figures and Tables

**Table 1:** Atomic concentrations in the ALD NiO_x_ films determined via XPS.

Samples	T_dep_ (°C)	Ni (%)	O (%)	C (%)	Ni/O Ratio
As-deposited	150	51.1	45.7	3.2	1.12
	200	52.3	44.8	2.9	1.17
	250	52.3	45.1	2.6	1.16
Post-annealed	250	52.1	45.5	2.4	1.15

## Data Availability

The data presented in this study are contained within the article.

## Supplementary Materials

The following supporting information can be downloaded at: https://www.mdpi.com/article/10.3390/nano15070474/s1, Figure S1: AFM surface morphologies of the NiO_x_ films with different deposition temperatures from 100 °C to 350 °C; Figure S2: SEM images of the NiO_x_ films with different deposition temperatures from 100 °C to 350 °C; Figure S3: XPS spectra of (a) wide scan survey and (b) C 1s of the as-deposited NiO_x_ film before and after Ar^+^ sputter surface etching; Figure S4: (a) UV–Vis spectra and (b,c) Tauc plots of (αhν)^2^ vs. hν for the ALD NiO_x_ films before and after annealing; Table S1: Comparison of the overpotential and chronoamperometric response of different NiO_x_-based OER catalysts.
