# Supplementary material for: Atomic Layer Deposition of Nickel Oxides as Electrocatalyst for Oxygen Evolution Reaction
Source: Nanomaterials (Basel). 2025 Mar 21;15(7):474. doi: 10.3390/nano15070474 (PMC11990215; doi:10.3390/nano15070474)
Supplement: Supplementary file 1 [file nanomaterials-15-00474-s001.zip › nanomaterials-3503782-supplementary.pdf]

Supplementary Materials

Atomic Layer Deposition of Nickel Oxides as Electrocatalyst for Oxygen Evolution Reaction

Jueyu Chen [1], Ruijie Dai [1], Hongwei Ma [1], Zhijie Lin [1], Yuanchao Li [1,*] and Bin Xi [1,*]

[1] School of Materials Science and Engineering, Key Laboratory for Polymeric Composite and Functional Materials of Ministry of Education, Sun Yat-sen University, Guangzhou 510006, China

* Correspondence: xibin3@mail.sysu.edu.cn (B.X.); liyuanchao@mail.sysu.edu.cn (Y.L.)

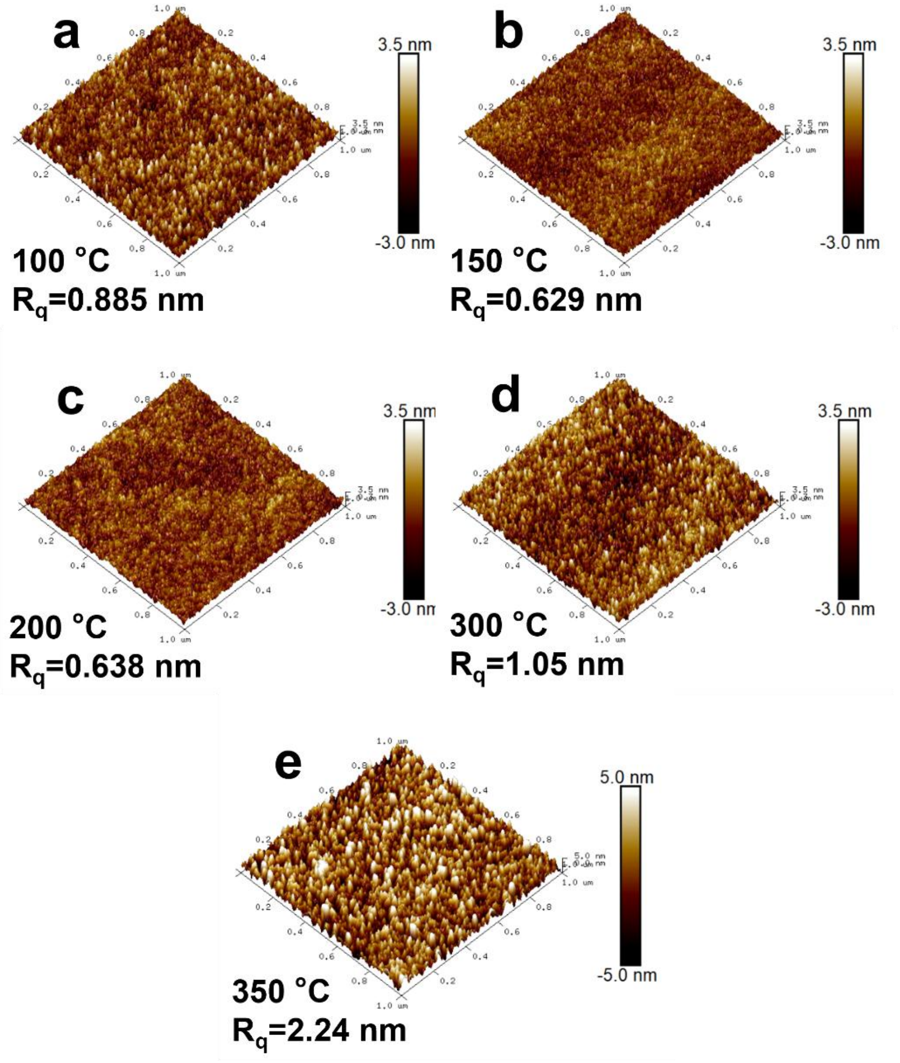

Figure S1. AFM surface morphologies of the NiO_x films with different deposition temperatures from 100 °C to 350 °C. The deposition cycle was 400 cycles.

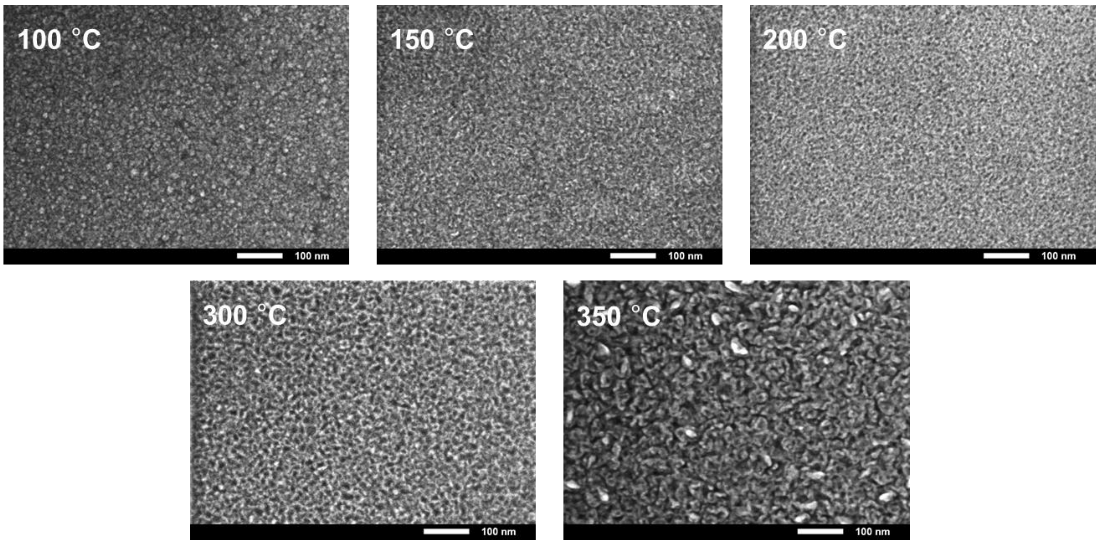

Figure S2. SEM images of the NiO_x films with different deposition temperatures from 100 °C to 350 °C. The deposition cycle was 400 cycles.

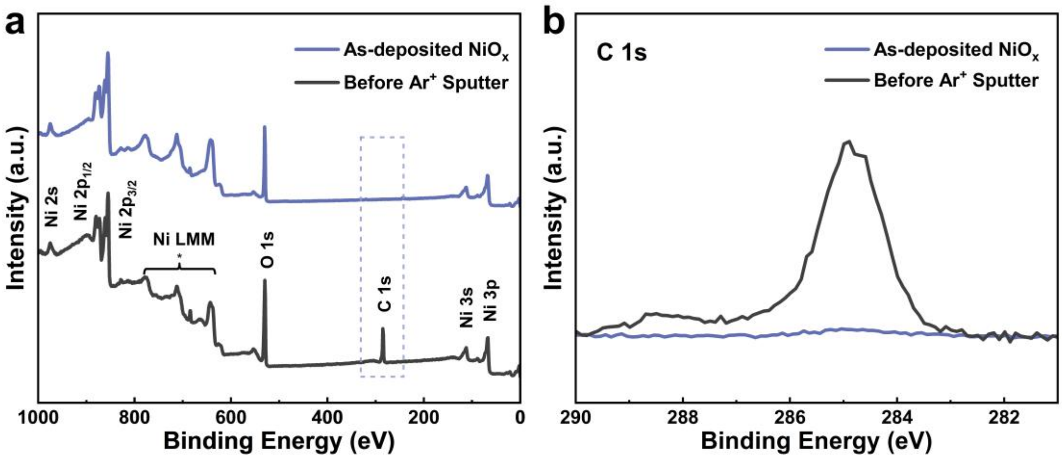

Figure S3. XPS spectra of a) wide scan survey and b) C 1s of the as-deposited NiO_x film before and after Ar^+ sputter surface etching.

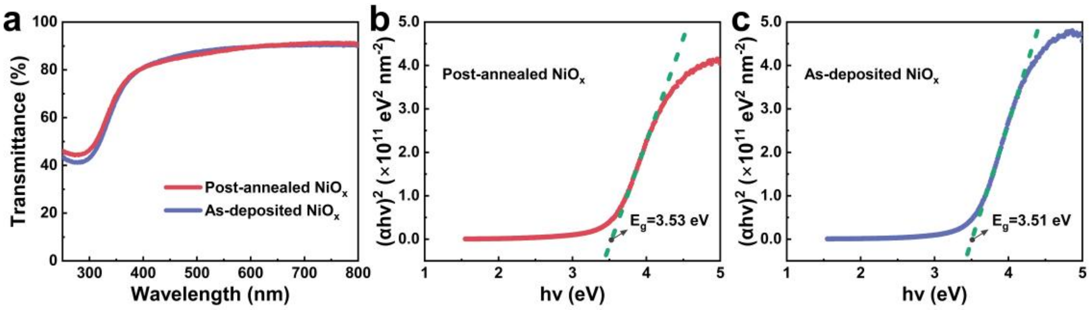

Figure S4. (a) UV-Vis spectra and (b, c) Tauc plots of $(\alpha h\nu)^2$ vs hν for the ALD NiO_x films before and after annealing. The films were deposited on glass substrate.

Table S1. Comparison of the overpotential and chronoamperometric response of different NiO_x-based OER catalysts.

Catalyst	Method	Electrolyte	Overpotential (at 10 mA cm^{-2})	Chronoamperometry
NiO/FTO[a]	ALD	1 M KOH	0.54 V	2.5 h
NiO/FTO	ALD	Fe^{3+}-saturated 0.1 M NaOH	0.36 V	-
NiO_x/SS[b]	ALD (this work)	1 M KOH	0.32 V	100 h
nanosphere NiO	Hydrothermal method	0.1 M KOH	0.61 V	2.78 h
NiO/NF[c]	Hydrothermal method	1 M KOH	0.35 V	14 h
NiO/NF	Solution combustion	pH = 13	0.35 V	25 h
Ni/NiO nanoparticles	Pulsed laser ablation	1 M KOH	0.31 V	12 h

[a]FTO refers to the fluorine-doped tin oxide coated glass substrate.
[b]SS refers to the stainless steel mesh substrate.
[c]NF refers to the nickel foam substrate.
